# 25-hydroxyvitamin D and increased all-cause mortality in very old women: the Newcastle 85+ study

**DOI:** 10.1111/joim.12273

**Published:** 2014-06-20

**Authors:** A Granic, T Aspray, T Hill, K Davies, J Collerton, C Martin-Ruiz, T von Zglinicki, T B L Kirkwood, J C Mathers, C Jagger

**Affiliations:** 1 Institute for Ageing and Health, Newcastle UniversityNewcastle upon Tyne, UK; 2 Human Nutrition Research Centre, Newcastle UniversityNewcastle upon Tyne, UK; 3 School of Agriculture, Food and Rural Development, Newcastle UniversityNewcastle upon Tyne, UK

**Keywords:** ageing, cohort study, mortality, risk factor, vitamins, women's health

## Abstract

**Objective:**

To investigate the associations between low and high concentrations of baseline serum 25-hydroxyvitamin D [25(OH)D] and all-cause mortality in very old (≥85 years) men and women over 6 years.

**Design, setting and subjects:**

Prospective mortality data from 775 participants in the Newcastle 85+ Study were analysed for survival in relation to 25(OH)D (season-specific quartiles and predefined cut-off values) and sex using Cox proportional hazards models. The models were fitted to the entire and restricted (nonusers of vitamin D-containing supplements and medication) cohorts.

**Results:**

For the entire cohort, mortality was higher in both the lowest and highest 25(OH)D season-specific quartiles [SQ1: hazard ratio (HR) 1.31, 95% confidence interval (CI) 1.01–1.69, *P* = 0.04; SQ4: HR 1.44, 95% CI 1.12–1.85, *P* = 0.004] compared with the combined middle quartiles (SQ2 + SQ3), after adjustment for sociodemographic factors. The increased risk for the highest quartile remained significant after further adjustment for lifestyle variables (SQ4: HR 1.37, 95% CI 1.06–1.77, *P* = 0.02) and was seen only in women in sex-specific analyses. Similarly, in sensitivity analyses with predefined 25(OH)D cut-off values, the highest 25(OH)D concentration (≥75 nmol L^−1^) was associated with a 2.4-fold increased risk of mortality in women (restricted cohort) after adjusting for all covariates.

**Conclusion:**

Low and high season-specific 25(OH)D quartiles were associated with increased risks of mortality over 6 years in the very old; this effect was particularly noticeable in women, including those who reported taking vitamin D-containing supplements/medication.

## Introduction

In the past two decades, accumulated evidence from cellular, animal and population-based studies has indicated the involvement of vitamin D metabolites in immunomodulation, cancer inhibition and cardiovascular, respiratory, brain and muscle function [1–5]. These extra-skeletal effects of vitamin D suggest its potential role in overall health and survival [6]. Recent observational studies in the general and older populations (≥65) have shown a non-linear relationship between serum hydroxyvitamin D [25(OH)D], the major circulatory and storage form of vitamin D, and both disease-specific and all-cause mortality [7–12]. This indicates that moderate rather than low or high concentrations of 25(OH)D may result in more favourable health outcomes and increased survival. Using an evidence-based approach for bone health, the US Institute of Medicine (IOM) has produced its latest report stating that: (i) concentrations of 50 nmol L^−1^ (20 ng mL^−1^) 25(OH)D meet the requirements of 97.5% of the North American population; (ii) concentrations of ≥75 nmol L^−1^ (30 ng mL^−1^) are not consistently associated with increased health benefits; and (iii) not all persons have inadequate 25(OH)D if concentrations are below 50 nmol L^−1^ [13]. Amongst at-risk groups, older adults are more likely to have lower 25(OH)D levels [14–16] because of reduced skin 7-dehydrocholesterol concentrations (the cutaneous precursor of vitamin D), inefficient renal activation of 25(OH)D and a reduction in outdoor activities with advancing age [17]. These factors also contribute to greater variability in both serum 25(OH)D concentrations and in the average requirement for vitamin D supplementation in older adults [13,18]. The findings of observational studies, randomized control trials (RCTs) and benefit–risk assessments all suggest that vitamin D supplementation in the general and older populations can ameliorate suboptimal 25(OH)D concentrations without adverse effects on disease-specific or all-cause mortality [19–24].

However, there is no agreement amongst researchers and healthcare professionals about the optimal, beneficial and age-specific 25(OH)D concentrations in relation to extra-skeletal outcomes and mortality [13,17,18,25,26], especially in older adults. Current evidence supports an inverse or non-linear association between 25(OH)D levels and mortality amongst adults aged 65 years and older. For example, a recent meta-analysis including 24 000 participants from nine prospective observational studies demonstrated a 25% increased pooled hazard ratio for all-cause mortality in the lowest compared with the highest 25(OH)D category in those aged ≥65 years [27]. A similar meta-analysis which included 12 studies (30 000 participants) confirmed an inverse association between 25(OH)D and mortality and a decrease in mortality risk of 8% for an increase in 25(OH)D of 20 nmol L^−1^ [28]. Two recent population-based studies from Denmark [10] and Israel [11], which both included over 40% of older adults (aged ≥65 years), showed a reversed J- and U-shaped relationship between 25(OH)D concentration and total mortality, respectively, and the best survival for individuals with 25(OH)D levels between 50 and 90 nmol L^−1^. Similarly, an examination of the National Health and Nutrition Examination Survey (NHANES) data (2001–2004) revealed no significant reduction in mortality above 21 ng mL^−1^ (52.6 nmol L^−1^) 25(OH)D [9].

To our knowledge, no prospective cohort study has investigated the relationship between 25(OH)D and mortality in the very old (aged ≥85 years), despite this being the fastest growing segment of many populations worldwide. Furthermore, except for the study conducted amongst members of the Clalit Health Services in Israel [11], the numbers of very old adults included in the above-mentioned studies were small. We therefore used data from the Newcastle 85+ Study to assess the relationship between serum 25(OH)D and all-cause mortality over 6 years in this elderly population.

## Methods

### Study sample

Participants were members of the Newcastle 85+ Study, a prospective study of health trajectories in a 1921 birth cohort recruited at the age of 85 through general practices in Newcastle and North Tyneside, UK. The study design has been described in detail elsewhere [29,30]. Briefly, a health assessment (comprising questionnaires, measurements, function tests and a fasting blood sample) was carried out in each participant's usual residence, including institutions, by a research nurse. General practice medical records were reviewed for diagnosed diseases, consultations and prescribed medication. At baseline in 2006/2007, both health assessment and general practice records data were available for 845 participants (58.2% of those eligible to participate) and general practice record review only for a further 188. The representativeness of the 845 eligible individuals with respect to the population of England and Wales of the same age has been reported [30]. Fasting blood samples were collected between July 2006 and September 2007 for 778 participants, and were sent within 1 h to the clinical biochemistry laboratory at the Royal Victoria Infirmary, Newcastle, UK for analyses.

### Ethics

The study was approved by the Newcastle & North Tyneside Local Research Committee One.

### Mortality data

Dates of death were obtained through the Health and Social Care Information Service UK. Survival time (in years) was calculated from the date of blood collection to the date of death or of censoring on 1 April 2012.

### Serum 25(OH)D assay and categorization

Serum 25(OH)D was measured with a radioimmunoassay kit (DiaSorin Corporation, Stillwater, MN, USA) using 25(OH)D-specific antibodies and ^125^I-labelled 25(OH)D as a tracer, as previously described [31]. The working range of the assay was 6–250 nmol L^−1^ and the interassay coefficients of variation were 8.4% and 12.6% at 25(OH)D concentrations of 39.4 nmol L^−1^ and 133.5 nmol L^−1^, respectively. In 775 (99.6%) participants, 25(OH)D concentrations were successfully measured at baseline and were categorized into season-specific quartiles (SQ1–SQ4) as shown in Table S1. Briefly, SQ1 ranged from 5–17 nmol L^−1^ (spring) to 8–30 nmol L^−1^ (autumn); SQ2 ranged from 18–26 nmol L^−1^ (spring) to 29–45 nmol L^−1^ (summer); SQ3 ranged from 27–46 nmol L^−1^ (spring) to 46–68 nmol L^−1^ (summer); and SQ4 ranged from ≥47 nmol L^−1^ (spring) to ≥69 nmol L^−1^ (summer). The middle quartiles (SQ2 and SQ3) were combined and used as the referent, thus forming three season-specific 25(OH)D groups: lowest (SQ1), middle (SQ2 + SQ3) and highest (SQ4). The dates of blood collection were categorized into winter (December–February), spring (March–May), summer (June–August) and autumn (September–November), and used to control for seasonal variation in 25(OH)D concentration in analyses with predefined 25(OH)D cut-off values (see below).

### Other measures and confounders

Confounders commonly used in studies investigating mortality risk in relation to 25(OH)D were considered for inclusion in models [7–12]. A detailed description of each confounder can be found in Table S2. Briefly, sociodemographic factors (sex, education, marital status and number of sources of income), lifestyle factors (smoking, alcohol intake and physical activity), morbidity (number of chronic diseases, waist–hip ratio and renal impairment) and mental health variables (global cognitive impairment assessed by the 30-point Standardized Mini Mental Status Examination (SMMSE) and depressive symptoms assessed by the 15-item Geriatric Depression Scale) were included in the models. The analyses were conducted for the entire cohort with available 25(OH)D data, and subsequently stratified by sex. We considered the intake of vitamin D-containing supplements and prescribed medication as important biological determinants of 25(OH)D status in this population [23,24] and conducted separate analyses with a restricted cohort [i.e. excluding 150 (19.4%) individuals who were taking vitamin D supplements/medication] with and without stratification by sex. Because of a low proportion of missing values (≤5%), confounders with missing data were imputed based on the mean (i.e. waist–hip ratio) or referent category (e.g. education or depressive symptoms). We used multiple linear regression to assess the confounders for multicollinearity and by inspecting VIF tolerance, eigenvalues and the condition index.

### Statistical analysis

The characteristics of participants who died before 1 April 2012 and those who survived thereafter were compared using independent *t*-tests and Mann–Whitney and chi-squared tests for continuous, ordered and categorical variables, respectively. We also compared those who had missing 25(OH)D data with those for whom 25(OH)D data were available. The Kaplan–Meier test was used to assess the relative risk of mortality by 25(OH)D categories in the entire and restricted cohorts and separately by sex. The time interval was calculated as period between the date of blood collection and the date of death or 1 April 2012. The Kaplan–Meier plots were inspected for proportionality of risk in relation to 25(OH)D categories over 6 years.

Cox proportional hazards models were employed to explore the relative risk of mortality associated with the season-specific 25(OH)D group across four models, adjusting for potential mortality- and vitamin D-related confounders. Log-minus-log plots were inspected for violation of the proportionality-of-hazard assumption by 25(OH)D group during follow-up. We estimated hazard ratios (HRs) with 95% confidence intervals (CIs) as: (i) unadjusted (Model 1); (ii) adjusted for sociodemographic variables (sex, education, marital status and income; Model 2); (iii) additionally adjusted for lifestyle factors (smoking, alcohol intake and physical activity; Model 3); and (iv) further adjusted for mental health and morbidity-related variables [cognitive impairment (≤25 points on the SMMSE scale), depressive symptoms, number of chronic diseases, renal impairment and waist–hip ratio (in tertiles)] (Model 4). Separate models were fitted to the entire and restricted cohorts, first with sex as a confounder and secondly stratified by sex.

We examined the potential non-linear relation between 25(OH)D (continuous) and all-cause mortality nonparametrically with restricted cubic splines [32] using SAS version 9.3 (SAS Institute Inc., Cary, NC, USA). The remaining analyses were performed using IBM SPSS Statistics software version 21 (IBM Corporation, Armonk, NY, USA).

### Sensitivity analysis

We compared the survival data obtained from analyses in which serum 25(OH)D was divided into season-specific quartiles (i.e. ‘data driven’) with results from the same models but in which 25(OH)D was categorized by predefined cut-off values [13,17] as follows: (i) <25 nmol L^−1^ (severely deficient), ≥25 to <50 nmol L^−1^ (deficient), 50–74 nmol L^−1^ (insufficient) and ≥75 nmol L^−1^ (sufficient) [17], with two middle (collapsed) categories as the referent; and (ii) as suggested by the IOM [13]: <30 nmol L^−1^ (deficient), 30–50 nmol L^−1^ (insufficient; referent) and >50 nmol L^−1^ (sufficient). Additionally, the final Cox proportional hazards model (Model 4) was further adjusted for the Fried frailty status (robust/pre-frail/frail), which was available for 552 participants (65.3%) [33]. We also assessed mortality risk approximately 1 year after baseline 25(OH)D measurement and excluded 77 (9.1%) participants who died before 1 January 2008.

## Results

The baseline characteristics of all participants and stratified by sex are shown in Table S3.

Compared with participants with successfully measured baseline 25(OH)D status (*n* = 775), those with missing 25(OH)D data (*n* = 70; 8.3%) were more likely to be women [χ^2^(1) = 8.7, *P* = 0.003], to have cognitive impairment [χ^2^(1) = 6.7, *P* = 0.01] and to be less physically active (U = 21 914.0, *P* < 0.001), and were less likely to drink alcohol [χ^2^(1) = 7.6, *P* = 0.006]. Participants with 25(OH)D data were more likely not to take any vitamin D-containing supplements but to take other vitamins [χ^2^(2) = 8.4, *P* = 0.02].

### All-cause mortality associated with season-specific 25(OH)D quartiles

At the end of follow-up (1 April 2012), approximately 6 years from baseline, 443 (52.4%) participants were still alive, and the mean survival time was 4.34 years (95% CI 4.20–4.48). Overall, 179 (58.9%) of 304 men with 25(OH)D status died during follow-up compared with 229 (48.6%) of 471 women. Those who died were more likely to be cognitively impaired at baseline (χ^2^(1) = 51.1, *P* < 0.001), to have renal impairment (χ^2^(1) = 9.6, *P* = 0.002), to smoke (χ^2^(1) = 7.2, *P* = 0.03), not to drink alcohol (χ^2^(1) = 11.2, *P* = 0.001) and not to take any vitamin supplements (χ^2^(2) = 6.8, *P* = 0.03), but to take prescribed vitamin D medication (χ^2^(1) = 18.1, *P* < 0.001).

**Fig 1:**
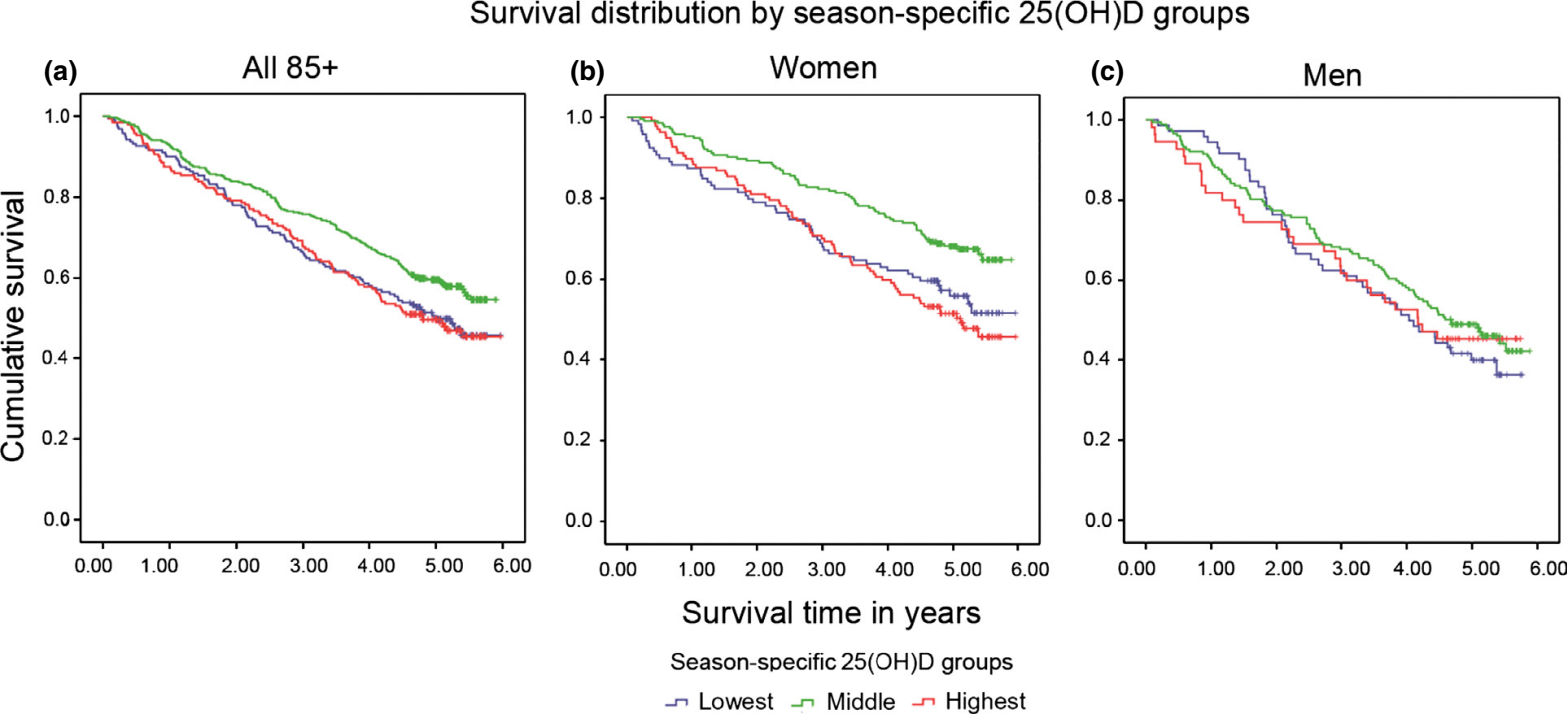
Kaplan–Meier plot of the probability of survival by season-specific 25-hydroxyvitamin D [25(OH)D] groups. There were statistically significant differences in survival across season-specific 25(OH)D groups (derived from season-specific quartiles) over 6 years of mortality follow-up amongst participants in the Newcastle 85+ Study. Individuals in the middle season-specific 25(OH)D group had a higher probability of survival compared with those in the two other groups (a). Survival was significantly longer in women (b) but not men (c) in the middle season-specific 25(OH)D group compared with women and men, respectively, in the two other groups.

In the unadjusted model, we found a significant association between 25(OH)D and survival in the entire cohort (Mantel-Cox χ^2^(2) = 7.90, *P* = 0.02), with the longest survival time in the middle season-specific group (SQ2 + SQ3: mean 4.51 years, 95% CI 4.32–4.69) and the shortest survival in highest group (SQ4: mean 4.12 years, 95% CI 3.84–4.41) (Model 1). Differences between the middle and two other 25(OH)D season-specific groups emerged within the first year of mortality follow-up and persisted until the last year (Fig. 1a).

This U-shaped relationship between survival and 25(OH)D remained after adjustment in a Cox proportional hazards model for sociodemographic variables (Table 1, left panel), with increased mortality in the lowest (SQ1: HR 1.31, 95% CI 1.01–1.69, *P* = 0.04) and the highest season-specific quartiles (SQ4: HR 1.44, 95% CI 1.12–1.85, *P* = 0.004) of 25(OH)D compared with the middle combined (referent) quartiles (Model 2). The U-shaped association disappeared after additional adjustment for lifestyle factors (smoking, alcohol intake and physical activity) with only participants in the highest quartile being at increased risk of mortality (SQ4: HR 1.37, 95% CI 1.06–1.77, *P* = 0.02) compared with the reference group (Model 3). This effect was further attenuated to a nonsignificant trend when adjusted for mental health and morbidity-related factors (SQ4: HR 1.25, 95% CI 0.97–1.63, *P* = 0.09; Model 4).

**Table 1:** Hazard ratios (HRs) for all-cause mortality by season-specific serum 25-hydroxyvitamin D [25(OH)D] groups (derived from season-specific quartiles) in the Newcastle 85+ Study

	Mortality in entire cohort (*n*_total_ = 775; *n*_cases_ = 363)			Mortality in restricted cohort^a^ (*n*_total_ = 625; *n*_cases_ = 272)		
	
Model	25(OH)D group^b^	HR (95% CI)	*P* value	25(OH)D group	HR (95% CI)	*P* value
Model 1	Lowest (*n*_cases_ = 97)	1.33 (1.03–1.71)	0.03	Lowest (*n*_cases_ = 96)	1.44 (1.11–1.87)	0.006
	Middle (*n*_cases_ = 166)	1 (reference)		Middle (*n*_cases_ = 139)	1 (reference)	
	Highest (*n*_cases_ = 100)	1.36 (1.06–1.74)	0.02	Highest (*n*_cases_ = 37)	1.02 (0.71–1.46)	0.93
Model 2	Lowest	1.31 (1.01–1.69)	0.04	Lowest	1.47 (1.12–1.91)	0.005
	Middle	1 (reference)		Middle	1 (reference)	
	Highest	1.44 (1.12–1.85)	0.004	Highest	1.04 (0.72–1.49)	0.85
Model 3	Lowest	1.15 (0.89–1.48)	0.30	Lowest	1.30 (0.99–1.70)	0.06
	Middle	1 (reference)		Middle	1 (reference)	
	Highest	1.37 (1.06–1.77)	0.02	Highest	1.11 (0.77–1.60)	0.57
Model 4	Lowest	1.10 (0.85–1.42)	0.48	Lowest	1.22 (0.93–1.60)	0.16
	Middle	1 (reference)		Middle	1 (reference)	
	Highest	1.25 (0.97–1.63)	0.09	Highest	1.05 (0.73–1.53)	0.79

Model 1 is unadjusted; Model 2 is adjusted for sociodemographic variables (sex, education, marital status and number of income sources); Model 3 is additionally adjusted for lifestyle factors (smoking, alcohol intake and physical activity); Model 4 is additionally adjusted for mental health and morbidity-related variables [depressive symptoms, cognitive impairment (≤25 points on the Standardized Mini Mental Status Examination), number of chronic diseases, renal impairment and waist–hip ratio. The following missing values were imputed with the reference (ref) category: education (*n* = 11, ref: 12–20 years), number of income sources (*n* = 5, ref: 4–5), marital status (*n* = 2, ref: married), physical activity (*n* = 6, ref: high), smoking status (*n* = 2, ref: former smoker), alcohol intake (*n* = 4, ref: yes), depressive symptoms (*n* = 42, ref: no), cognitive status at baseline (*n* = 2, ref: cognitively impaired), renal impairment (*n* = 1, ref: yes). *n*_total_, total number of participants; *n*_cases_, number of deaths; CI, confidence interval.

a Analyses were restricted to the cohort of individuals not taking vitamin D supplements/prescribed medication.

b Middle two quartiles of season-specific serum 25(OH)D were combined and served as the reference (see Supplementary Table 1 for details).

When the analyses were restricted to those not taking vitamin D either as supplements or prescribed medication (restricted cohort), and after adjustment for sociodemographic variables (Model 2), shorter survival was evident only in the lowest 25(OH)D quartile (SQ1: HR 1.47, 95% CI 1.12–1.91, *P* = 0.005), which was attenuated to a trend (*P* = 0.06) after adjustment for lifestyle factors, and abrogated in the fully adjusted model (Table 1, right panel).

In unadjusted sex-stratified analyses of the entire cohort, survival was longest in the middle season-specific 25(OH)D group in women (mean 4.86 years, 95% CI 4.63–5.08; Mantel-Cox χ^2^(2) = 13.15, *P* = 0.001) but not in men (mean 4.07 years, 95% CI 3.78–4.36; Mantel-Cox χ^2^(2) = 1.01, *P* = 0.6) (Fig. 1b,c). Similar results were found in the fully adjusted model for the entire cohort with a shorter survival in the highest season-specific quartile (compared with the middle referent quartiles) but again only in women (SQ4: HR 1.51, 95% CI 1.06–2.14, *P* = 0.02; Model 4) (Table 2, left panel). In the restricted cohort only, women in the lowest season-specific quartile had an increased risk of mortality (SQ1: HR 1.77, 95% CI 1.19–2.64, *P* = 0.005) after adjusting for sociodemographic and lifestyle variables (Model 3), which was attenuated but remained significant in the fully adjusted model (SQ1: HR 1.63, 95% CI 1.07–2.49, *P* = 0.02; Model 4) (Table 2, right panel). The finding that the association between raised 25(OH)D concentration and increased mortality was only observed in women, and furthermore was not found in the restricted cohort, suggests that the increased mortality amongst all participants in the highest season-specific 25(OH)D quartile may be attributed to women taking vitamin D-containing supplements/medication.

**Table 2:** Hazard ratios (HRs) for all-cause mortality by season-specific serum 25-hydroxyvitamin D [25(OH)D] groups (derived from season-specific quartiles) for men and women in the Newcastle 85+ Study

	25(OH)D group^a^	HR (95% CI)	*P* value	25(OH)D group	HR (95% CI)	*P* value
	
Model	Mortality in men (entire cohort, *n*_total_ = 304, *n*_cases_ = 169)			Mortality in women (entire cohort, *n*_total_ = 471, *n*_cases_ = 194)		
Model 1	Lowest (*n*_cases_ = 44)	1.20 (0.84–1.71)	0.33	Lowest (*n*_cases_ = 53)	1.57 (1.10–2.25)	0.01
	Middle (*n*_cases_ = 95)	1 (reference)		Middle (*n*_cases_ = 71)	1 (reference)	
	Highest (*n*_cases_ = 30)	1.10 (0.73–1.66)	0.64	Highest (*n*_cases_ = 70)	1.78 (1.28–2.48)	<0.001
Model 2	Lowest	1.10 (0.76–1.58)	0.63	Lowest	1.60 (1.12–2.29)	0.01
	Middle	1 (reference)		Middle	1 (reference)	
	Highest	1.08 (0.71–1.64)	0.73	Highest	1.87 (1.34–2.62)	<0.001
Model 3	Lowest	0.96 (0.66–1.39)	0.82	Lowest	1.42 (0.99–2.04)	0.06
	Middle	1 (reference)		Middle	1 (reference)	
	Highest	1.13 (0.74–1.73)	0.56	Highest	1.59 (1.13–2.24)	0.008
Model 4	Lowest	0.95 (0.65–1.39)	0.79	Lowest	1.27 (0.87–1.84)	0.22
	Middle	1 (reference)		Middle	1 (reference)	
	Highest	1.06 (0.69–1.64)	0.78	Highest	1.51 (1.06–2.14)	0.02

	Mortality in men (restricted cohort^b^, *n*_total_ = 276, *n*_cases_ = 150)			Mortality in women (restricted cohort^b^, *n*_total_ = 349, *n*_cases_ = 122)		
Model 1	Lowest (*n*_cases_ = 44)	1.24 (0.86–1.77)	0.25	Lowest (*n*_cases_ = 52)	1.91 (1.30–2.81)	0.001
	Middle (*n*_cases_ = 88)	1 (reference)		Middle (*n*_cases_ = 51)	1 (reference)	
	Highest (*n*_cases_ = 18)	0.81 (0.49–1.35)	0.42	Highest (*n*_cases_ = 19)	1.38 (0.82–2.34)	0.23
Model 2	Lowest	1.15 (0.79–1.66)	0.47	Lowest	1.98 (1.34–2.93)	0.001
	Middle	1 (reference)		Middle	1 (reference)	
	Highest	0.79 (0.47–1.32)	0.37	Highest	1.37 (0.80–2.34)	0.25
Model 3	Lowest	1.01 (0.69–1.48)	0.95	Lowest	1.77 (1.19–2.64)	0.005
	Middle	1 (reference)		Middle	1 (reference)	
	Highest	0.90 (0.53–1.51)	0.68	Highest	1.37 (0.80–2.35)	0.26
Model 4	Lowest	0.95 (0.64–1.40)	0.78	Lowest	1.63 (1.07–2.49)	0.02
	Middle	1 (reference)		Middle	1 (reference)	
	Highest	0.90 (0.53–1.54)	0.71	Highest	1.32 (0.76–2.28)	0.32

Model 1 is unadjusted; Model 2 is adjusted for sociodemographic variables (education, marital status and number of income sources); Model 3 is additionally adjusted for lifestyle factors (smoking, alcohol intake and physical activity); Model 4 is additionally adjusted for mental health and morbidity-related variables [depressive symptoms, cognitive impairment (≤25 points on the Standardized Mini Mental Status Examination), number of chronic diseases, renal impairment and waist – 31 – hip ratio (in tertiles)]. The following missing values were imputed with the reference (ref) category: education (*n* = 11, ref: 12–20 years), number of income sources (*n* = 5, ref: 4–5), marital status (*n* = 2, ref: married), physical activity (*n* = 6, ref: high), smoking status (*n* = 2, ref: former smoker), alcohol intake (*n* = 4, ref: yes), depressive symptoms (*n* = 42, ref: no), cognitive status at baseline (*n* = 2, ref: cognitively impaired) and renal impairment (*n* = 1, ref: yes). *n*_total_, total number of participants; *n*_cases_, number of deaths; CI, confidence interval.

a Middle two quartiles of season-specific serum 25(OH)D were combined and served as the reference.

b Analyses were restricted to the cohort of individuals not taking vitamin D supplements/prescribed medication.

**Fig 2:**
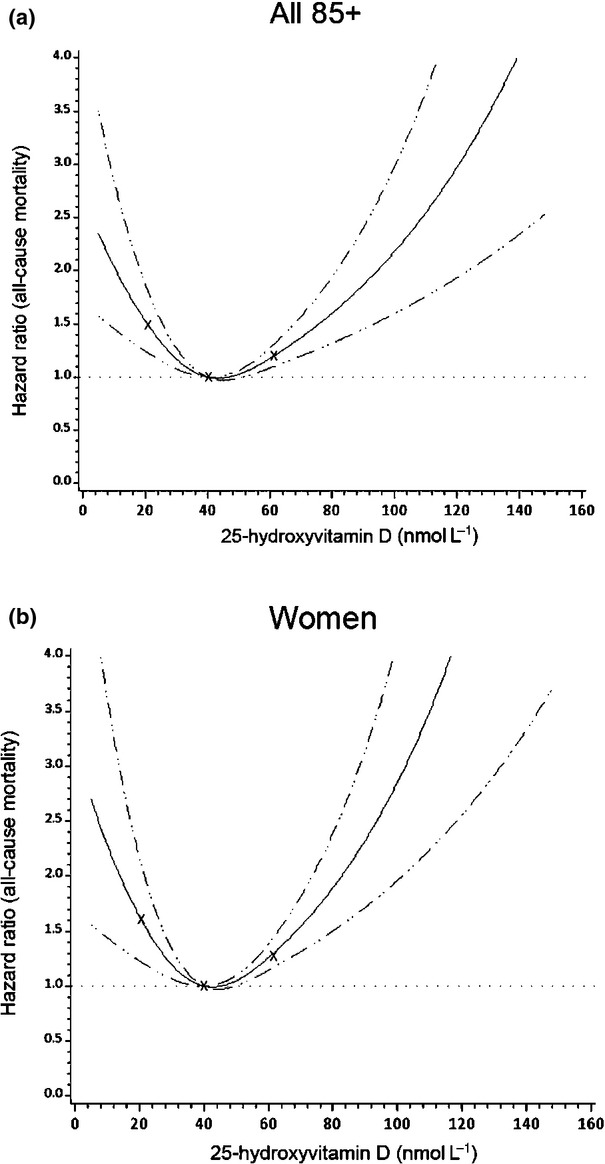
Restricted cubic spline curves of dose–response relationship between serum 25-hydroxyvitamin D [25(OH)D] and all-cause mortality in the Newcastle 85+ Study. A non-linear dose–response relation was observed in the entire cohort (a) and in women (b) using restricted cubic splines with three knots fitted at percentiles (at approximately 20, 40 and 60 nmol L^−1^) (test for non-linear relation, *P* < 0.001).

For the restricted cubic splines analysis, three, four and five knots were fitted at percentiles with three knots [at approximately 20, 40 and 60 nmol L^−1^ 25(OH)D] having a marginally better fit as judged by the Akaike Information Criterion. A non-linear dose–response relation between 25(OH)D concentration and all-cause mortality was evident in the entire cohort (test for non-linear relation, *P* < 0.001; Fig. 2a) and in women (test for non-linear relation, *P* < 0.001; Fig. 2b).

### Sensitivity analyses

#### All-cause mortality associated with 25(OH)D predefined cut-off values

In the entire 25(OH)D cohort, we detected a similar U-shaped relationship between 25(OH)D and 6-year survival (Table S4), with both ‘severely deficient’ (<25 nmol L^−1^) and ‘sufficient’ (≥75 nmol L^−1^) 25(OH)D concentration ranges [17] being associated with increased risks of mortality (43% and 86%, respectively), compared with the combined middle concentration ranges after adjusting for season of blood testing and sociodemographic confounders (Model 2). Further adjustment for lifestyle and mental health and morbidity-related variables (Model 4) abolished the risk in those with concentrations <25 nmol L^−1^ and reduced the risk in the ≥75 nmol L^−1^ group to 51% (*P* = 0.006). However, in contrast to analyses with 25(OH)D categories defined by season-specific quartiles, women but not men with a concentration of 25(OH)D ≥75 nmol L^−1^ had significantly increased mortality in the entire and restricted cohorts, even in the fully adjusted models (Table S5).

Proportionality assumptions of 25(OH)D groups were confirmed although inspection of log-minus-log plots of all models. The results were unchanged when models were fitted excluding imputed values for covariates (data not shown).

We excluded participants who died within the first year of mortality follow-up (by 1 January 2008) and found similar results. In the fully adjusted model (Model 4), increased mortality was observed among women in the highest season-specific quartile (SQ4: HR 1.56, 95% CI 1.06–2.30, *P* = 0.02) and in those in the ≥75 nmol L^−1^ group (HR 1.91, 95% CI 1.25–2.91, *P* = 0.003) compared with the middle groups. In the restricted cohort, women with a concentration of 25(OH)D ≥75 nmol L^−1^ had a 2.8-fold increased risk of dying (data not shown).

In analyses in which the IOM cut-off values for 25(OH)D status were used [13], the U-shaped relationship with all-cause mortality was not observed (Tables S6 and S7). Women with a concentration of 25(OH)D >50 nmol L^−1^ in the entire but not in the restricted cohort had a 50% increased risk of mortality (*P* = 0.03) after adjusting for season of blood collection and sociodemographic variables (Model 2), which remained raised (42% increased risk) but was no longer significant in the fully adjusted model (*P* = 0.07).

To explore the role of frailty in the association between 25(OH) status and mortality, the final model (Model 4) was additionally adjusted for the Fried frailty status, with the covariate physical activity excluded because of correlation and overlap of concepts between the two variables. Women belonging to the highest season-specific quartile or ‘sufficient’ 25(OH)D category (i.e. ≥75 nmol L^−1^) had an increased risk of mortality compared with the corresponding (referent) group (SQ4: HR 1.73, 95% CI 1.08–2.77, *P* = 0.02; ‘sufficient’ group: HR 2.22, 95% CI 1.32–3.75, *P* = 0.003) (data not shown). This suggests that higher concentrations of 25(OH)D may adversely affect survival in women irrespective of their frailty status.

## Discussion

In this prospective cohort study of older adults aged ≥85 years, we found a dose–response relationship between serum 25(OH)D and all-cause mortality, with both the lowest and highest season-specific 25(OH)D quartiles being associated with higher mortality over 6 years. The higher risk of mortality amongst participants with the highest concentrations [a threshold range of ≥47 nmol L^−1^ (spring) to ≥69 nmol L^−1^ (summer) for the highest season-specific quartile] appeared to be driven largely by women taking vitamin D-containing supplements and/or prescribed medication. Using 25(OH)D as a continuous covariate and fitting with restricted cubic splines confirmed the relationship between 25(OH)D and mortality. Moreover, in sensitivity analyses using predefined 25(OH)D cut-off values [17], the highest (≥75 nmol L^−1^) ‘sufficient’ concentration was still associated with an increased risk of mortality amongst women who were not using vitamin D supplements/medication, suggesting that at this concentration the risk of reduced survival is not solely due to supplementation. These results remained when we excluded participants who died within the first year of follow-up to control for the possibility that poor health and reduced mobility close to the time of death affected 25(OH)D status. Furthermore, the greater risk of mortality amongst women with the highest 25(OH)D concentrations (SQ4 or ‘sufficient’ categories) was independent of their frailty status [34].

To our knowledge, this is the first observational study to suggest a U-shaped relationship between serum 25(OH)D and all-cause mortality in very old adults. Several recent systematic reviews and meta-analyses of prospective cohort studies investigating the association between 25(OH)D status and risk of mortality [27,28,35,36] have demonstrated a shorter survival amongst adults with the lowest (<25 or <50 nmol L^−1^) compared with highest 25(OH)D concentrations, especially in those aged 65 years and older [27,36]. In other studies, a non-linear relationship was noted with favourable survival outcomes at concentrations between 50 and 90 nmol L^−1^ [9–11]. However, except for the study conducted in Israel [11], relatively few participants aged over 85 years were included in these studies.

Our findings are in general agreement with those of a large retrospective study from general practices in Copenhagen (CopD Study) [10] showing an inverse J-shaped relationship between 25(OH)D and mortality, with the longest survival at concentrations of 50–60 nmol L^−1^ during 3 years of follow-up. Similarly, a historical prospective study of more than 420 000 members of the Clalit Health Services in Israel [11], which included >20 000 participants aged ≥85 years, found that the lowest risk of mortality and acute coronary syndrome was associated with 25(OH)D in the range of 20–36 ng mL^−1^ (50–90 nmol L^−1^) during 4.5 years of follow-up. A meta-analysis of 14 prospective cohort studies involving the general population (age range 45–80 years and 1.3–27.0 years of follow-up) also suggested a non-linear relationship between 25(OH)D and mortality, but 25(OH)D levels of ∼75–87.5 nmol L^−1^ were considered optimal [35].

We observed statistically significant sex differences in mean (SD) 25(OH)D concentration which were lower than recommended (i.e. below 50 nmol L^−1^) for optimal bone health in both men and women [13], especially in our restricted cohort [38.48 (21.39) nmol L^−1^]. The intake of vitamin D supplements/medication is an important biological determinant of 25(OH)D status in this cohort [23,24], and therefore we included intake of supplements/medication and as potential confounders. The higher mortality rates observed amongst very old women, with higher 25(OH)D concentrations [SQ4 or ‘sufficient’ (≥75 nmol L^−1^) categories] whether users or nonusers of vitamin D supplements/medication, respectively, have not been reported previously [20–24]. The Women's Health Initiative calcium/vitamin D RCT, a 7-year combined therapy intervention (1 g calcium and 400 IU vitamin D daily), reported a trend towards mortality reduction amongst postmenopausal women aged <70 years, but neither a beneficial nor an adverse effect in women aged >70 years [22]. The latest meta-analysis of 56 RCTs of vitamin D supplementation and survival [24] demonstrated a decrease in all-cause mortality amongst predominantly older adults including women aged ≥70 years, but also found adverse renal outcomes associated with vitamin D_3_ and calcium combination therapy.

Lower (<37.5 nmol L^−1^) and higher (≥75 nmol L^−1^) concentrations of 25(OH)D have been moderately associated with frailty amongst older women (aged ≥69 years) in the Study of Osteoporotic Fractures [34], and the risk of death was significantly increased amongst frail NHANES III participants (aged ≥60 years) in the lowest (<49.5 nmol L^−1^) compared with not frail participants in the highest (>84.1 nmol L^−1^) 25(OH)D quartiles [37]. In the present study, those in the middle 25(OH)D quartiles/groups were least likely to be frail (*P* < 0.001); however, the increased risk of mortality in women in the lowest and highest 25(OH)D groups was independent of their frailty status and was not affected by exclusion of those who died within first year of follow-up. Compared with men, women were more likely to have osteoporosis (*P* < 0.001) and to be treated with prescribed vitamin D medication (*P* < 0.001), which included calcium combination therapy (Table S2). Because the duration and dosage of the therapy was not known, we cannot exclude any potential adverse effects of co-supplementation, or the possibility that the higher mortality amongst individuals taking vitamin D supplements/medication and with a level of 25(OH)D ≥75 nmol L^−1^ could be driven partly by those with a prior long-standing vitamin D deficiency corrected recently by supplementation.

The results from observational cohort studies exploring sex differences in mortality in relation to 25(OH)D are inconclusive and have not included the very old. The NHANES III showed a U-shaped relationship between 25(OH)D levels and mortality in the general population of women (aged ≥20 years) but not in men at concentrations of <50 and >125 nmol L^−1^ [12]. In a study of older men (a birth cohort from 1920 to 1924, aged 71 at baseline) from the Uppsala region, an increased risk of total and cancer mortality was observed at both low (<46 nmol L^−1^) and high (>98 nmol L^−1^) 25(OH)D concentrations over 12.7 years of follow-up [7]. In both these studies, the longest survival was associated with the middle 25(OH)D categories, but the thresholds were much higher than in the present study (NHANES III: 75–100 nmol L^−1^; Uppsala Study of Older Men: 46–98 nmol L^−1^); this difference may be related to the age of participants, habitual diet, supplementation, length of follow-up or other factors/covariates.

Several limitations of our study should be noted. There remains the possibility of residual confounding by additional factors that affect the relationship between serum 25(OH)D and mortality. On the other hand, having a large number of variables in a fully adjusted model (Model 4) may have contributed to a nonsignificant or biased result, given the fact that we categorized 25(OH) into season-specific quartiles (*n*_average_ - mean number per quartile = 194), and therefore may have had limited power for detection of associations. Although we controlled for the number of chronic diseases and for frailty status, increased mortality amongst older women may be mediated by other mechanisms associated with nonoptimal 25(OH)D levels such as polypharmacy [38] or an acute inflammatory response [39,40]. Recent studies have demonstrated a rapid decline in serum 25(OH)D after elective hip or knee surgery or after an acute inflammatory insult, thus 25(OH)D may be an unreliable biomarker of vitamin D status up to 3 months after the event [39,40]. Although using quartiles of 25(OH)D may limit the impact of hazard risks, we confirmed the U-shaped relationship between 25(OH)D and mortality using predefined cut-off values [17] and restricted cubic splines. Whilst recognizing that there is seasonal variations in 25(OH)D status, the results were based on a single measurement, which may misclassify 25(OH)D levels throughout the year. Additionally, lower values of 25(OH)D in the present study could be due to the choice of assay (DiaSorin), which has been reported to provide lower readings compared with other methods (e.g. liquid chromatography tandem-mass spectrometry) [41], and to overestimate vitamin D deficiency/insufficiency (<30 nmol L^−1^/<50 nmol L^−1^, respectively), especially in older women aged ≥65 years [42]. We used physical activity as a proxy for exposure to ultraviolet-B radiation, a major source of circulating 25(OH)D, and did not control for dosage or duration of exposure for vitamin D supplements/medication, or for their potential interaction with other medication [38]. Whether or not there was any long-standing vitamin D deficiency amongst women with the highest 25(OH)D concentrations, which may have been corrected by supplementation prior to baseline, was unknown. The results from this study may be generalized to elderly white populations (aged ≥85 years) living at similar latitudes. We did not explore disease-specific mortality in relation to 25(OH)D because of high rates of multimorbidity in this age group [30].

The strengths of our study include: (i) the homogenous age of the cohort; (ii) the prospective 6-year follow-up; (iii) inclusion of several mortality-related covariates in both the entire and restricted cohorts; (iv) analyses stratified by sex and exposure (i.e. intake of vitamin D supplements/medication) and (v) use of ‘data driven’ season-specific 25(OH)D cut-off values as the preferred method of adjusting for seasonal variability in vitamin D status [43].

In summary, both low and high concentrations of 25(OH)D were associated with an increased risk of all-cause mortality amongst very old adults, especially amongst women who reported taking vitamin D-containing supplements and prescribed medication as well as in women with concentrations ≥75 nmol L^−1^ irrespective of intake of supplements/medication. Future recommendations for optimal concentrations of serum 25(OH)D should pay special attention to the very old, to prevent problems related to over-treatment in this age group [13,18,25,26].

## Funding

This study was supported by the National Institute for Health Research Newcastle Biomedical Research Centre based at Newcastle Hospitals Foundation Trust and Newcastle University (AG). The Newcastle 85+ Study has been funded by the Medical Research Council, Biotechnology and Biological Sciences Research Council and the Dunhill Medical Trust. The authors have also been supported by the British Heart Foundation, Unilever Corporate Research, Newcastle University and the National Health Service (NHS) North of Tyne (Newcastle Primary Care Trust). The views expressed are those of the authors and not necessarily those of the NHS, UK.

## Conflict of interest statement

No conflicts of interest to declare.

## Supporting Information

**Table S1.** Season-specific serum 25(OH)D^a^ quartile cut-offs in the Newcastle 85+ Study. **Table S2**. Description of confounders used in Cox proportional hazard models. **Table S3**. Characteristics of participants in the Newcastle 85+ Study. **Table S4.** HRs for all-cause mortality by predefined 25(OH)D cut-offs [17] in the Newcastle 85+ Study. **Table S5.** HRs for all-cause mortality by predefined 25(OH)D cut-offs [17] for men and women in the Newcastle 85+ Study. **Table S6.** HRs for all-cause mortality by serum 25(OH)D predefined cut-offs [13] in the Newcastle 85+ Study. **Table S7**.HRs for all-cause mortality by serum 25(OH)D predefined cut-offs [13] in women in the Newcastle 85+ Study.
