# Supplementary material for: 25-hydroxyvitamin D and increased all-cause mortality in very old women: the Newcastle 85+ study
Source: J Intern Med. 2014 Jun 20;277(4):456–67. doi: 10.1111/joim.12273 (PMC4406141; doi:10.1111/joim.12273)
Supplement: Supplementary file 1 [file joim0277-0456-sd1.docx]

**R1 Supplementary Tables**

Supplementary Table 1 Season-specific serum 25(OH)D^a^ quartile cut-offs in the Newcastle 85+ Study

__________________________________________________________________________________________________________________

Season-specific quartiles Summer Autumn Winter Spring

(Jun-Aug) (Sep-Nov) (Dec-Feb) (Mar-May)

*n* = 312 (40.1%) *n* = 180 (23.1%) *n* = 165 (21.2%) *n* = 122 (15.7%) __________________________________________________________________________________________________________________

SQ1 (n_total_ = 191 [24.6%]) 5-28 nmol/L 8-30 nmol/L 6-22 nmol/L 5-17 nmol/L

SQ2 (n_total_ = 199 [25.7%]) 29-45 nmol/L 31-43 nmol/l 23-32 nmol/L 18-26 nmol/L

SQ3 (n_total_ = 193 [24.9%]) 46-68 nmol/L 44-61 nmol/L 33-59 nmol/L 27-46 nmol/L

SQ4 (n_total_ = 192 [24.8%]) ≥69 nmol/L ≥62 nmol/L ≥60 nmol/L ≥47 nmol/L

___________________________________________________________________________________________________________________

^a^Season-specific serum 25(OH)D quartiles (SQ1 to SQ4) were created for each season of blood draw to adjust for seasonal variation of

UVB exposure and 25(OH)D skin production. A combined season-specific quartiles were recoded into season-specific 25(OH)D groups,

and middle quartiles (SQ2+SQ3) were combined into a middle (referent) group.

Supplementary Table 2 Description of confounders used in Cox proportional hazard models

_____________________________________________________________________________

Confounder Description and coding

____________________________________________________________________________________

*Sociodemographic variables*

sex (categorical) women/men (0/1)

years of education (ordinal) 0-9/10-11/≥12 years

marital status (categorical) single/widowed, divorced or separated/married (0/1/2)

sources of income (ordinal) 0-2/3/4-5 sources which include state retirement pension,

occupational pension, private pension, savings/investments, and welfare benefits

*Mental health and morbidity-related variables*

Cognitive status (Standardized Mini-Mental normal (≥26 SMMSE points)/impaired (≤25 SMMSE

Status Examination [SMMSE])^a^ (categorical) points) (0/1)

Geriatric Depression Scale (GDS)^b^ (ordinal) scores 0-5 (no depressive symptoms), 6-7 (mild

depression), ≥8 (severe depression) (0/1/2)

waist-hip ratio (ordinal in tertiles with cut-offs calculated as waist (cm)/hip (cm) and imputed with means

of 0.59, 0.85, and 0.91) for males and females for 97 individuals with missing

values; (0/1/2)

number of chronic diseases (ordinal) included total number of following diseases: arthritis (e.g.

osteoarthritis, rheumatoid arthritis, spondylosis, etc.), hypertension, cardiac disease (e.g. heart failure, angina, myocardial infarction, coronary angioplasty or stent, coronary artery bypass graft), respiratory disease

(e.g. COPD, asthma, bronchiectasis, pulmonary fibrosis,

Cerebrovascular disease (e.g. stroke, transient ischaemic attack, carotid endarterectomy, etc.), diabetes (Type 1, Type 2, and unspecified), cancer (any cancer diagnosis in the past 5 years excluding non- melanoma skin cancer); categorized as 0-1, 2 and ≥2 (0/1/2)

renal impairment (dichotomous) yes/no (1/0) diagnosis determined by the Chronic Kidney

Disease Epidemiology Collaboration (CKD-EPI)

guidelines^c^

*Lifestyle variables*

physical activity (categorical) low/moderate/high (0/1/2)

smoking status (categorical) never smoker/ former smoker/ current smoker (0/1/2)

current alcohol intake (dichotomous) yes/no (1/0)

*Serum 25(OH)D status variables*

season of blood collection (categorical) June-August (Summer)/September-November (Autumn)/

December-February (Winter)/March-May (Spring)

(0/1/2/3)

vitamin D-containing supplements (categorical) yes, at least one/no but taking other vitamin supplements/not taking any vitamin supplements (0/1/2); ‘taking others’ included non-prescribed multivitamins, multivitamins with minerals, and combination of vitamins A and C with other vitamins except D

prescribed vitamin D medication (dichotomous) yes/no (1/0), ‘yes’ included prescription vitamin D, calcium with vitamin D, bisphosphonate with calcium and vitamin D, and strontium with calcium and vitamin D

____________________________________________________________________________________

^a^Molloy DW, Standish TI. A guide to standardize Mini-Mental State Examination. *Int Psychogeriatr* 1997; **9** (**Suppl 1**): 87-94.

^b^Yasavage JA. Geriatric Depression Scale. *Psychopharmacol Bull* 1988; **24**: 709-11.

^c^[Stevens LA](http://www.ncbi.nlm.nih.gov/pubmed?term=Stevens%20LA%5BAuthor%5D&cauthor=true&cauthor_uid=17591522), [Manzi J](http://www.ncbi.nlm.nih.gov/pubmed?term=Manzi%20J%5BAuthor%5D&cauthor=true&cauthor_uid=17591522), [Levey AS](http://www.ncbi.nlm.nih.gov/pubmed?term=Levey%20AS%5BAuthor%5D&cauthor=true&cauthor_uid=17591522), *et al*. Impact of creatinine calibration on performance of GFR estimating equations in a pooled individual patient database. [*Am J Kidney Dis*](http://www.ncbi.nlm.nih.gov/pubmed/17591522) 2007; **50**: 21-35.

Supplementary Table 3 Characteristics of participants in the Newcastle 85+ Study

____________________________________________________________________________________________________________________

Characteristic All Men Women *P* value^a^

*n* = 845 *n* = 319 *n* = 526

____________________________________________________________________________________________________________________

*Serum 25(OH)D status variables*

25(OH)D M (SD) nmol/L 45.43 (26.30) 42.88 (22.70) 47.07 (28.28) 0.03

Season-specific 25(OH)D quartiles % (*n*) 0.01

Lowest (SQ1) 24.6 (191) 23.7 (72) 25.3 (119)

Middle^b^ (SQ2+SQ3) 50.6 (392) 58.2 (177) 45.6 (215)

Highest (SQ4) 24.8 (192) 18.1 (55) 29.1 (137)*

Season of blood testing % (*n*) NS

December-February 21.2 (165) 19.4 (59) 22.3 (106)

March-May 15.7 (122) 16.1 (49) 15.4 (73)

June-August 40.1 (312) 39.1 (119) 40.6 (193)

September-November 23.1 (180) 25.3 (77) 21.7 (103)

Vitamin D supplements % (*n*) NS

Yes 3.8 (32) 2.2 (7) 4.8 (25)

No but taking other supplements 37.4 (316) 35.4 (113) 38.6 (203)

Not taking any supplements 58.8 (497) 62.4 (199) 56.7 (298)

Vitamin D prescribed medication % (*n*) <0.001

Yes 16.5 (139) 7.5 (24) 21.9 (115)

*Sociodemographic variables*

Marital status % (*n*) <0.001

Single 8.2 (69) 5.7 (18) 9.7 (51)

Widowed/divorced/separated 61.7 (519) 41.6 (132) 73.9 (387)

Married 30.1 (253) 52.7 (167)* 16.4 (86)

Years of education % (*n*) NS

0-9 y 64.4 (534) 62.3 (195) 65.7 (339)

10-11 y 22.8 (189) 24.6 (77) 21.7 (112)

Supplementary Table 3 Characteristics of men and women in the Newcastle 85+ Study (continued)

____________________________________________________________________________________________________________________

≥12 y 12.8 (106) 13.1 (41) 12.6 (65)

Number of sources of income % (n) <0.001

0-2 30.3 (254) 20.5 (65) 36.3 (189)

3 46.7 (391) 53.9 (171) 42.2 (220)

4-5 23.0 (193) 25.6 (81) 21.5 (112)

*Lifestyle variables*

Physical activity % (n) <0.001

Low 23.3 (189) 20.9 (65) 24.8 (124)

Moderate 43.0 (349) 32.5 (101) 49.5 (248)

High 33.7 (274) 46.6 (145) 25.7 (129)

Smoking status % (n) <0.001

Never 35.8 (301) 25.6 (81) 42.0 (220)

Current smoker 5.7 (48) 4.4 (14) 6.5 (34)

Former smoker 58.5 (491) 69.9 (221)* 51.5 (270)

Current alcohol intake % (n) <0.001

Yes 59.5 (488) 76.3 (238) 49.2 (250)

*Mental health variables*

Global cognitive status^c^ % (n) NS

Normal (≥26 SMMSE points) 71.4 (599) 71.9 (228) 71.1 (371)

Impaired (≤25 SMMSE points) 28.6 (240) 28.1 (89) 28.9 (151)

Depressive symptoms^d^ % (n) 0.02

0-5/none 78.9 (607) 83.4 (247) 76.1 (360)

6-7/mild 12.7 (98) 9.5 (28) 14.8 (70)

≥8/severe 8.3 (64) 7.1 (21) 9.1 (43)

*Morbidity-related variables*

Number of chronic diseases % (n) NS

0-1 29.1 (246) 29.2 (93) 29.1 (153)

2 30.1 (254) 31.7 (101) 29.1 (153)

≥3 40.8 (345) 39.2 (125) 41.8 (220)

Supplementary Table 3 Characteristics of men and women in the Newcastle 85+ Study (continued) ______________________________________________________________________________________________________________________

Renal impairment % (n) NS

Yes 23.9 (185) 22.4 (68) 24.8 (117)

Waist-hip ratio tertiles^e^ % (n) <0.001

Lowest 30.1 (255) 7.8 (25) 43.7 (230)

Middle 36.6 (308) 27.9 (89) 41.6 (219)

Highest 33.4 (282) 64.3 (205) 14.6 (77)

______________________________________________________________________________________________________________________

^a^Independent t-test for continuous and Mann-Whitney U for ordered and χ^2^ test for categorical variables. ^b^Serum 25(OH)D was categorized in season-specific quartiles. Two middle quartiles were combined to create ‘middle’ group. ^c^Global cognitive impairment was defined as scoring ≤25 points on Standardized Mini Mental Status Exam (SMMSE). ^d^Fifteen points geriatric depression scale (GDS). ^e^Cut-offs of 0.85 and 0.91.

*In the post hoc χ^2^ test, adjusted residuals were inspected to determine which cell contributed the most to rejecting the null hypothesis at α=0.005.

Supplementary Table 4 HRs for all-cause mortality by predefined 25(OH)D cut-offs [17] in the Newcastle 85+ Study

____________________________________________________________________________________________________________________

Mortality in entire cohort (*n_total_* = 775, n_cases_ = 363) Mortality in restricted cohort^a^ (*n_total_*  = 625, n_cases_ = 272)

________________________________________________________________________________________________________

Model 25(OH)D group^b^ HR (95% CI) *P* value 25(OH)D group HR (95% CI) *P* value

____________________________________________________________________________________________________________________

Model 1 Lowest (<25 nmol/L) 1.42 (1.11-1.80) 0.005 Lowest (<25 nmol/L) 1.53 (1.19-1.97) 0.001

Middle (25-74 nmol/L) 1 (reference) Middle (25-74 nmol/L) 1 (reference)

Highest (≥75 nmol/L) 1.69 (1.28-2.22) <0.001 Highest (≥75 nmol/L) 1.44 (0.91-2.28) 0.12

Model 2 Lowest (<25 nmol/L) 1.43 (1.11-1.84) 0.005 Lowest (<25 nmol/L) 1.63 (1.25-2.13) <0.001

Middle (25-74 nmol/L) 1 (reference) Middle (25-74 nmol/L) 1 (reference)

Highest (≥75 nmol/L) 1.86 (1.40-2.46) <0.001 Highest (≥75 nmol/L) 1.50 (0.94-2.40) 0.09

Model 3 Lowest (<25 nmol/L) 1.21 (0.94-1.56) 0.14 Lowest (<25 nmol/L) 1.36 (1.04-1.79) 0.03

Middle (25-74 nmol/L) 1 (reference) Middle (25-74 nmol/L) 1 (reference)

Highest (≥75 nmol/L) 1.67 (1.25-2.23) 0.001 Highest (≥75 nmol/L) 1.55 (0.96-2.48) 0.07

Model 4 Lowest (<25 nmol/L) 1.19 (0.92-1.53) 0.19 Lowest (<25 nmol/L) 1.31 (0.99-1.73) 0.06 Middle (25-74 nmol/L) 1 (reference) Middle (25-74 nmol/L) 1 (reference)

Highest (≥75 nmol/L) 1.51 (1.27-2.03) 0.006 Highest (≥75 nmol/L) 1.54 (0.95-2.49) 0.08

____________________________________________________________________________________________________________________ ^a^Analyses were restricted to cohort not taking vitamin D supplements and prescribed medication. ^b^ Predefined cut-offs were used to create three 25(OH)D groups [17]. Kaplan-Meier analysis revealed the longest but similar mean survival times in middle categories, which were combined to create middle 25(OH)D group (referent).

Model 1 is unadjusted.

Model 2 is adjusted for season of blood testing and sociodemographic variables (sex, education, marital status, and number of income sources).

Model 3 is additionally adjusted for lifestyle factors (smoking, alcohol intake, and physical activity).

Model 4 is additionally adjusted for mental health and morbidity-related variables (depressive symptoms, cognitive impairment [≤25 SMMSE points], number of chronic diseases, renal impairment, and waist-hip ratio [in tertiles]).

The following missing values were imputed with the reference category: education (*n* = 11, ref: 12-20 years), number of income sources (*n* = 5, ref: 4 to 5), marital status (*n* = 2, ref: married), physical activity (*n* = 6, ref: high), smoking status (*n* = 2, ref: former smoker), alcohol intake (*n* = 4, ref: yes), depressive symptoms (*n* = 42, ref: no), cognitive status at baseline (*n* = 2, ref: cognitively impaired), renal impairment (*n* = 1, ref: yes).

Supplementary Table 5 HRs for all-cause mortality by predefined 25(OH)D cut-offs [17] for men and women in the Newcastle 85+ Study

____________________________________________________________________________________________________________________

Model 25(OH)D group^b^ HR (95% CI) *P* value 25(OH)D group HR (95% CI) *P* value

________________________________________________________________________________________________________

Mortality in **men** (entire cohort, *n_total_*  = 304, n_cases_ = 169) Mortality in **women** (entire cohort, *n_total_*  = 471, n_cases_ = 194)

____________________________________________________________________________________________________________________

Model 1 Lowest (<25 nmol/L) 1.50 (1.06-2.13) 0.02 Lowest (<25 nmol/L) 1.47 (1.05-2.06) 0.03

Middle (25-74 nmol/L) 1 (reference) Middle (25-74 nmol/L) 1 (reference)

Highest (≥75 nmol/L) 1.47 (0.86-2.42) 0.13 Highest (≥75 nmol/L) 2.08 (1.47-2.94) <0.001

Model 2 Lowest (<25 nmol/L) 1.48 (1.02-2.15) 0.04 Lowest (<25 nmol/L) 1.44 (1.02-2.05) 0.04

Middle (25-74 nmol/L) 1 (reference) Middle (25-74 nmol/L) 1 (reference)

Highest (≥75 nmol/L) 1.49 (0.90-2.49) 0.12 Highest (≥75 nmol/L) 2.19 (1.53-3.14) <0.001

Model 3 Lowest (<25 nmol/L) 1.21 (0.83-1.78) 0.32 Lowest (<25 nmol/L) 1.30 (0.92-1.86) 0.14 Middle (25-74 nmol/L) 1 (reference) Middle (25-74 nmol/L) 1 (reference)

Highest (≥75 nmol/L) 1.39 (0.81-2.36) 0.23 Highest (≥75 nmol/L) 1.85 (1.28-2.68) 0.001

Model 4 Lowest (<25 nmol/L) 1.21(0.82-1.80) 0.34 Lowest (<25 nmol/L) 1.18 (0.82-1.69) 0.38

Middle (25-74 nmol/L) 1 (reference) Middle (25-74 nmol/L) 1 (reference)

Highest (≥75 nmol/L) 1.27 (0.72-2.22) 0.41 Highest (≥75 nmol/L) 1.72 (1.19-2.51) 0.004

_________________________________________________________________________________________________________

Mortality in **men** (restricted cohort, *n_total_* = 276, n_cases_ = 150) Mortality in **women** (restricted cohort, *n_total_* = 349, n_cases_ = 122)

_____________________________________________________________________________________________________________________

Model 1 Lowest (<25 nmol/L) 1.56 (1.09-2.22) 0.02 Lowest (<25 nmol/L) 1.77 (1.22-2.57) 0.003

Middle (25-74 nmol/L) 1 (reference) Middle (25-74 nmol/L) 1 (reference)

Highest (≥75 nmol/L) 1.09 (0.57-2.09) 0.80 Highest (≥75 nmol/L) 2.00 (1.05-3.80) 0.04

Supplementary Table 5 HRs for all-cause mortality by serum 25(OH)D predefined cut-offs [17] for men and women in the Newcastle 85+ Study (continued)

_____________________________________________________________________________________________________________________

Model 2 Lowest (<25 nmol/L) 1.58 (1.08-2.30) 0.03 Lowest (<25 nmol/L) 1.76 (1.19-2.61) 0.005

Middle (25-74 nmol/L) 1 (reference) Middle (25-74 nmol/L) 1 (reference)

Highest (≥75 nmol/L) 1.20 (0.61-2.36) 0.60 Highest (≥75 nmol/L) 2.20 (1.06-4.15) 0.03

Model 3 Lowest (<25 nmol/L) 1.29 (0.87-1.91) 0.20 Lowest (<25 nmol/L) 1.60 (1.07-2.38) 0.02

Middle (25-74 nmol/L) 1 (reference) Middle (25-74 nmol/L) 1 (reference)

Highest (≥75 nmol/L) 1.28 (0.64-2.60) 0.49 Highest (≥75 nmol/L) 2.24 (1.11-4.50) 0.02

Model 4 Lowest (<25 nmol/L) 1.15 (0.77-1.73) 0.50 Lowest (<25 nmol/L) 1.46 (0.96-2.23) 0.08

Middle (25-74 nmol/L) 1 (reference) Middle (25-74 nmol/L) 1 (reference)

Highest (≥75 nmol/L) 1.25 (0.61-2.56) 0.54 Highest (≥75 nmol/L) 2.44 (1.20-5.00) 0.01

____________________________________________________________________________________________________________________

^a^Analyses were restricted to cohort not taking vitamin D supplements and prescribed medication. ^b^ Predefined cut-offs were used to create three 25(OH)D groups [17]. Kaplan-Meier analysis revealed the longest but similar mean survival time in middle categories, which were combined to create middle 25(OH)D group (referent).

Model 1 is unadjusted.

Model 2 is adjusted for season of blood testing and sociodemographic variables ( education, marital status, and number of income sources).

Model 3 is additionally adjusted for lifestyle factors (smoking, alcohol intake, and physical activity).

Model 4 is additionally adjusted for mental health and morbidity-related variables (depressive symptoms, cognitive impairment [≤25 SMMSE points], number of chronic diseases, renal impairment, and waist-hip ratio [in tertiles]).

The following missing values were imputed with the reference category: education (*n* = 11, ref: 12-20 years), number of income sources (*n* = 5, ref: 4 to 5), marital status (*n* = 2, ref: married), physical activity (*n* = 6, ref: high), smoking status (*n* = 2, ref: former smoker), alcohol intake (*n* = 4, ref: yes), depressive symptoms (*n* = 42, ref: no), cognitive status at baseline (*n* = 2, ref: cognitively impaired), renal impairment (*n* = 1, ref: yes).

Supplementary Table 6 HRs for all-cause mortality by serum 25(OH)D predefined cut-offs [13] in the Newcastle 85+ Study

____________________________________________________________________________________________________________________

Mortality in entire cohort (*n_total_* = 775, n_cases_ = 363) Mortality in restricted cohort^a^ (*n_total_* = 625, n_cases_ = 272)

________________________________________________________________________________________________________

Model 25(OH)D group^b^ HR (95% CI) *P* value 25(OH)D group HR (95% CI) *P* value

____________________________________________________________________________________________________________________

Model 1 Lowest (<30 nmol/L) 1.28 (0.99-1.66) 0.06 Lowest (<30 nmol/L) 1.33 (1.01-1.74) 0.04

Middle (30-50 nmol/L) 1 (reference) Middle (30-50 nmol/L) 1 (reference)

Highest (>50 nmol/L) 1.23 (0.95-1.59) 0.11 Highest (>50 nmol/L) 0.92 (0.67-1.28) 0.63

Model 2 Lowest (<30 nmol/L) 1.26 (0.96-1.64) 0.09 Lowest (<30 nmol/L) 1.37 (1.04-1.81) 0.03

Middle (30-50 nmol/L) 1 (reference) Middle (30-50 nmol/L) 1 (reference)

Highest (>50 nmol/L) 1.29 (1.00-1.67) 0.05 Highest (>50 nmol/L) 0.94 (0.67-1.31) 0.71

Model 3 Lowest (<30 nmol/L) 1.16 (0.89-1.51) 0.28 Lowest (<30 nmol/L) 1.26 (0.95-1.66) 0.11

Middle (30-50 nmol/L) 1 (reference) Middle (30-50 nmol/L) 1 (reference)

Highest (>50 nmol/L) 1.29 (0.99-1.68) 0.05 Highest (>50 nmol/L) 1.04 (0.74-1.45) 0.84

Model 4 Lowest (<30 nmol/L) 1.12 (0.89-1.52) 0.28 Lowest (<30 nmol/L) 1.25 (0.95-1.66) 0.12 Middle (30-50 nmol/L) 1 (reference) Middle (30-50 nmol/L) 1 (reference)

Highest (>50 nmol/L) 1.23 (0.95-1.60) 0.12 Highest (>50 nmol/L) 1.04 (0.74-1.45) 0.84

____________________________________________________________________________________________________________________ ^a^Analyses were restricted to cohort not taking vitamin D supplements and prescribed medication. ^b^ Predefined cut-offs were used to create three 25(OH)D groups [13].

Model 1 is unadjusted.

Model 2 is adjusted for season of blood testing and sociodemographic variables (sex, education, marital status, and number of income sources).

Model 3 is additionally adjusted for lifestyle factors (smoking, alcohol intake, and physical activity).

Model 4 is additionally adjusted for mental health and morbidity-related variables (depressive symptoms, cognitive impairment [≤25 SMMSE points], number of chronic diseases, renal impairment, and waist-hip ratio [in tertiles]).

The following missing values were imputed with the reference category: education (*n* = 11, ref: 12-20 years), number of income sources (*n* = 5, ref: 4 to 5), marital status (n = 2, ref: married), physical activity (*n* = 6, ref: high), smoking status (*n* = 2, ref: former smoker), alcohol intake (*n* = 4, ref: yes), depressive symptoms (*n* = 42, ref: no), cognitive status at baseline (*n* = 2, ref: cognitively impaired), renal impairment (*n* = 1, ref: yes).

Supplementary Table 7 HRs for all-cause mortality by serum 25(OH)D predefined cut-offs [13] in **women** in the Newcastle 85+ Study

_____________________________________________________________________________________________________________________

Mortality in women (entire cohort) (*n_total_* = 471, *n_cases_* = 194) Mortality in women (restricted cohort) (*n_total_* = 349, n_cases_ = 122)

_________________________________________________________________________________________________________

Model 25(OH)D group^b^ HR (95% CI) *P* value 25(OH)D group HR (95% CI) *P* value

_____________________________________________________________________________________________________________________

Model 1 Lowest (<30 nmol/L) 1.23 (0.84-1.79) 0.29 Lowest (<30 nmol/L) 1.31 (0.90-2.03) 0.15

Middle (30-50 nmol/L) 1 (reference) Middle (30-50 nmol/L) 1 (reference)

Highest (>50 nmol/L) 1.47 (1.03-2.10) 0.03 Highest (>50 nmol/L) 0.97 (0.58-1.60) 0.90

Model 2 Lowest (<30 nmol/L) 1.20 (0.81-1.76) 0.37 Lowest (<30 nmol/L) 1.31 (0.86-2.01) 0.21

Middle (30-50 nmol/L) 1 (reference) Middle (30-50 nmol/L) 1 (reference)

Highest (>50 nmol/L) 1.50 (1.05-2.15) 0.03 Highest (>50 nmol/L) 0.95 (0.57-1.58) 0.84

Model 3 Lowest (<30 nmol/L) 1.14 (0.77-1.67) 0.52 Lowest (<30 nmol/L) 1.26 (0.82-1.93) 0.29 Middle (30-50 nmol/L) 1 (reference) Middle (30-50 nmol/L) 1 (reference)

Highest (>50 nmol/L) 1.37 (0.95-1.97) 0.23 Highest (>50 nmol/L) 1.01 (0.60-1.71) 0.97

Model 4 Lowest (<30 nmol/L) 1.12 (0.75-1.66) 0.59 Lowest (<30 nmol/L) 1.27 (0.81-1.98) 0.30

Middle (30-50 nmol/L) 1 (reference) Middle (30-50 nmol/L) 1 (reference)

Highest (>50 nmol/L) 1.42 (0.98-2.06) 0.07 Highest (>50 nmol/L) 1.09 (0.64-1.87) 0.75

_____________________________________________________________________________________________________________________

^a^Analyses were restricted to women not taking vitamin D supplements and prescribed medication. ^b^Predefined cut-offs were used to create three 25(OH)D groups 25(OH)D groups [13].

Model 1 is unadjusted.

Model 2 is adjusted for season of blood testing and sociodemographic variables (education, marital status, and number of income sources).

Model 3 is additionally adjusted for lifestyle factors (smoking, alcohol intake, and physical activity).

Model 4 is additionally adjusted for mental health and morbidity-related variables (depressive symptoms, cognitive impairment [≤25 SMMSE points], number of chronic diseases, renal impairment, and waist-hip ratio [in tertiles]).

The following missing values were imputed with the reference category: education (*n* = 11, ref: 12-20 years), number of income sources (*n* = 5, ref: 4 to 5), marital status (*n* = 2, ref: married), physical activity (*n* = 6, ref: high), smoking status (*n* = 2, ref: former smoker), alcohol intake (*n* = 4, ref: yes), depressive symptoms (*n* = 42, ref: no), cognitive status at baseline (*n* = 2, ref: cognitively impaired), renal impairment (*n* = 1, ref: yes).
